# Site disparities in apoptotic variants as predictors of risk for second primary malignancy in patients with squamous cell carcinoma of the head and neck

**DOI:** 10.1186/s12885-016-2110-y

**Published:** 2016-02-08

**Authors:** Yan Sun, Wenbin Yu, Erich M. Sturgis, Wei Peng, Dapeng Lei, Qingyi Wei, Xicheng Song, Guojun Li

**Affiliations:** Department of Head and Neck Surgery, Unit 1445, The University of Texas MD Anderson Cancer Center, 1515 Holcombe Boulevard, Houston, TX 77030 USA; Department of Otorhinolaryngology and Head and Neck Surgery, Yuhuangding Hospital of Qingdao University, Yantai, China; Key Laboratory of Carcinogenesis and Translational Research(Ministry of Education/Beijing), Department of Head and Neck surgery, Peking University Cancer Hospital & Institute, Beijing, China; Department of Epidemiology, The University of Texas MD Anderson Cancer Center, Houston, TX 77030 USA; Department of Biostatistics and Human Genetics Center, University of Texas School of Public Health, 1200 Herman Pressler St, Houston, TX 77030 USA; Department of Otolaryngology, Qilu Hospital, Shandong University; Key Laboratory of Otolaryngology, Ministry of Health, P.R. China, Jinan, Shandong 250012 China; Duke Cancer Institute, Duke University Medical Center, Durham, NC 27710 USA

**Keywords:** *FAS/FASLG*, Squamous cell carcinoma of head and neck, Second primary malignancy, Susceptibility, Polymorphism, Oropharyngeal cancer, Nonoropharyngeal cancer

## Abstract

**Background:**

*FAS/FASL* promoter variants are considered in altering transcriptional activity of those genes and consequently alter regulation of cell death. However, no studies have investigated whether tumor sites contribute to the association between *FAS/FASL* polymorphisms and risk for second primary malignancy (SPM).

**Method:**

In this study, *FAS*670 A > G, *FAS*1377 G > A, *FASL*124 A > G, and *FASL*844C > T polymorphisms were genotyped in 752 OPC and 777 non-OPC patients. Both univariate and multivariable cox proportional hazard models were used to assess the associations.

**Results:**

The univariate and multivariable analyses showed that patients with index OPC and *FASL*844 CT/TT genotype had significantly increased risk of SPM (cHR, 2.5; 95 % CI, 1.1–5.8, *P =* 0.043 and aHR, 2.7; 95 % CI, 1.2–6.0, *P =* 0.032) compared with those with *FASL*844 CC genotype as the reference group, while index non-OPC patients with *FAS*670 AG/GG and *FasL*844 CT/TT genotypes had significantly increased risk of SPM (cHR, 2.2 and 1.8; 95 % CI, 1.2–5.7 and 1.1–3.2; and *P =* 0.04 and 0.041, respectively and aHR, 2.4 and 1.7; 95 % CI, 1.1–5.1 and 1.0-3.0; and *P =* 0.043 and 0.049, respectively) compared with their corresponding AA and CC genotypes . Moreover, patients carrying more *FAS/FASL* variants significantly increased risk of SPM among index non-OPC patients. The stratified analysis showed that smoking status differently modified the associations between *FAS/FASL* polymorphisms and risk of SPM among index non-OPC from OPC patients.

**Conclusion:**

These results suggested that *FAS/FASL* polymorphisms might significantly modify SPM risk among patients with SCCHN in a tumor site-specific manner.

## Background

The squamous cell carcinomas of the head and neck (SCCHN) arise in the upper aerodigestive tract including the oral cavity, the oropharynx, the hypopharynx, and the larynx [1]. The SCCHN are associated with a high incidence rate of second primary malignancy (SPM), for which the risk remains at a substantially high during the lifespan of the patients [2–5]. Although we have seen the declining trend of overall SCCHN incidence, the decrease in incidence of SPM is heterogeneous across all tumor sites within the head and neck regions.

The oropharyngeal cancer (OPC) has the features of an aggressive local tumor, with a moderately high recurrence rate, high frequency of SPM, and high frequency of medical comorbidities [6]. While the incidence of OPC is increasing, the attributable risk to SPM in patients with index OPC has declined since the 1990s. Moreover, the incidence of HPV-positive OPC cases increases among OPC patients, whereas the oropharynx is the subsite with the lowest SPM risk [3, 7–9]. Therefore, HPV is highly relevant to the SPM rates in OPC patients. The nonoropharyngeal cancer (non-OPC) includes tumors which occur in oral cavity, larynx, and hypopharynx. Compared with patients with OPC, the patients with non-OPC have the consistently high risk of SPM [3].

The SPMs in OPC and non-OPC patients still remain one of major factors that contribute to the poor prognosis of these patients [2, 10–12]. Cigarette smoking and alcohol use have been found to be associated with the risk of SPM [13, 14], while only a small proportion of these patients develop a SPM, suggesting that there is a person to person variation in genetic susceptibility to SPM among the patients [2]. We have previously reported that genetic polymorphisms involved in several molecular pathways were associated with the risk of SPM after primary SCCHN, such as carcinogen metabolism, DNA repair, and cell cycle control [12, 15–21].

Apoptosis is the physiological mechanism of programmed cell death that plays an important role in diverse biological processes such as development, homeostasis of tissues, and elimination of cancer cells [22, 23]. The acquired ability to resist apoptotic stimuli is one of the primary characteristics of a malignant cell, and abnormal regulation of apoptosis is a key mechanism in the development of cancer [24]. FAS is a cell surface receptor that can interact with FASLG to trigger apoptosis [25]. Therefore, FAS/FASLG pathway plays an important role in regulation of apoptosis and maintenance of cellular homeostasis, and genetic alteration of FAS/FASLG signaling pathway may result in immune escape, and thus tumorigenesis including SPM.

The two polymorphisms identified in the *FAS* promoter region: *FAS*1377 G > A rs2234767 and *FAS*670 A > G rs1800682 result in decreased promoter activity and decreased *FAS* gene expression in consequence [26]. Another two polymorphisms in *FASL* promoter: *FASL*844 C > T rs763110 and *FASL* 124 A > G rs5030772 may increase basal expression of *FASL*, resulting in aberrant FASL expression and subsequently increased susceptibility to cancer [26, 27].

Although we have previously reported a study on association of these functional polymorphisms with SPM risk among SCCHN patients, showing that *FAS*670 and *FASL*844 polymorphisms were associated with a significantly increased risk for SPM in overall SCCHN patients [28], no studies have evaluated the associations between these polymorphisms with risk of SPM in different tumor sites of SCCHN. Since *FAS/FASL* pathway plays a critical role in apoptosis, this study might provide novel information for prognosis and secondary as well as tertiary cancer prevention of these patients. Therefore, we evaluated the associations between each or in combination of the four *FAS/FASL* genetic variants and risk of SPM among 752 index OPC and 777 index non-OPC patients, respectively.

## Methods

### Study subjects

All the participants with incident cases were consecutively recruited between May 1995 and December 2010 through the Head and Neck Center at The University of Texas, M.D. Anderson Cancer Center. The inclusion criteria for cases were: (1) newly diagnosed, histopathologically confirmed squamous cell carcinomas of the oral cavity, oropharynx, hypopharynx, or larynx; (2) no history of treatment with either chemotherapy or radiation therapy treatment; and (3) patients with previous history of cancer excepting non-melanoma skin cancer, distant metastases at presentation, primary tumors of the nasopharynx or sinoasal tract, primary tumors outside the upper aerodigestive tract, cervical metastases of unknown origin, or histopathologic diagnoses other than squamous cell carcinoma were excluded. The study received approval from the institutional review boards of both MD Anderson and Kelsey-Seybold, and all study subjects signed an informed consent when approached for recruitment. All patients completed a standardized questionnaire including demographic, exposure information (including tobacco and alcohol), work history, and family history data, and donated a blood sample for genotyping data at the time of recruitment. Participants who were not able to provide their blood samples during the recruitment were excluded in the analysis.

Patients were monitored through their treatment and post-treatment course with regularly scheduled clinical and radiographic examinations. SPMs were distinguished from local recurrences based on modified criteria of Warren and Gates [29]. Second lesions with different histophathologic type and/or occurring >5 years following treatment for the primary tumor and/or clearly separated by normal epithelium based on clinical and radiographic assessment were considered SPM. The second lesion was classified as a local recurrence rather than a SPM if there was discrepancy or differing opinion about the origin of the tumor. Pulmonary lesions were considered SPM if they had a non-squamous histology or if they were isolated squamous lesions >5 years from initial SCCHN and felt to be SPM by the thoracic oncologist and thoracic surgeon. Medical record review for follow-up status of all patients was performed under supervision of the staff head and neck surgeon. Patients who had smoked >100 cigarettes in their lifetimes were categorized as “ever-smokers,” and others were “never-smokers.” Similarly, subjects who had drunk alcoholic beverages at least once a week for >1 year previously were as “ever-drinkers,” and others were “never-drinkers.” Clinical data were obtained at initial presentation and through follow-up examinations and included overall stage at presentation of index tumor, site of index tumor, and treatment. Index cancer stages were then dichotomized into the early stage (clinical stage I and II) and late stage (III and IV). Types of treatment were categorized into three groups: surgery only, surgery and chemotherapy and/or radiotherapy, and chemotherapy and/or radiotherapy.

### Genotyping

Genomic DNA was extracted from a leukocyte cell pellet for *FAS/FASL* genotyping, which was obtained from the buffy coat by the centrifugation of 1 mL of whole blood, using the Qiagen DNA Blood Mini Kit (Qiagen Inc.) in accordance with the manufacturer’s instructions. The genotyping of the four SNPs was performed as previously described [26]. Moreover, there was 100 % concordance when we randomly selected at least 10 % of the random samples to retest for confirmation of the original findings.

### Statistical analysis

The statistical analyses were performed by Intercooled Stata 13.0 (Stata Inc., College Station, TX). All tests were two-sided, and a P-value < 0.05 was considered the cutoff for statistical significance. All statistical tests were processed in patients with index OPC and in patients with index non-OPC separately. SPM was considered as the primary endpoint of this study. The Student’s t tests were used to compare the mean age and follow-up time of the patients who developed a SPM and those who did not. The differences in ethnicity, sex, smoking and alcohol status, index tumor stage, treatment, and genotype distributions between the two groups were evaluated using the chi-squared test. Time-to-event was calculated from the date of diagnosis of the index cancer patients to the date of SPM occurrence. Patients who did not know to have an event at the date of last contact, or who died were censored. The associations between individual epidemiological risk factors, clinical characteristics including index tumor stage and treatment variables, and time to the occurrence of SPMs, were initially assessed using univariate Cox proportional hazards regression models. The data were consistent with the assumptions of the Cox proportional hazards regression model from the examination of Kaplan-Meier survival curves. In the univariate and multivariable analyses (adjusted by age, gender, ethnicity, smoking and drinking status, index cancer stage, and treatment), the associations were calculated using hazard ratios (HR) and their 95 % confidence intervals (CI) for the development of the SPMs. For each polymorphism, the wild-type genotype was set as the reference group; the variant genotypes of each polymorphism were combined. In addition, for the combined analysis of the four polymorphisms, the patients with 0–2 risk genotypes as the low-risk group were used as the reference group in comparison with the group with 3–4 variant genotypes as the high-risk group.

## Results

### Study patients characteristics

The study subjects were a cohort of 1529 patients, including 752 patients with index OPC and 777 patients with non-OPC. There were significant differences in the median age at diagnosis and follow-up time between index OPC patients and index non-OPC patients (54 years *vs*. 59 years; *P =* 0.0011; 51.3 months; 63.8 months; *P =* 0.00012, respectively), and patients who developed SPMs were more likely to be index non-OPC (*P =* 0.001) when we compared the patients with and without SPM. Our data indicated that patients with index OPC had a lower rate of SPM compared to those with non-OPC (approximately 6 % *vs.* 11 %). Table 1 shows the demographics, risk factors, and clinical variables for the 752 patients with index OPC and 777 patients with non-OPC. For OPC patients, there were 710 (94.4 %) patients who did not develop SPM and 42 (5.6 %) patients who developed SPMs. The median age at diagnosis was 54 years (range, 28–84 years). In this patient cohort, we found age and smoking were significantly associated with SPM development (*P =* 0.004 for age and *P =* 0.010 for smoking), while we did not observe significant differences between patients with and without SPMs, regarding other characteristic variables. For the patients with index non-OPC, there were 695 (89.4 %) patients who did not develop SPMs and 82 (10.9 %) patients who developed SPMs. The median age at diagnose was 59 years (range, 18–94 years). We found that the patients who developed SPMs were more likely to be older (*P =* 0.024), ever smokers (*P =* 0.037), ever alcohol user (*P =* 0.005), non-Hispanic white (*P =* 0.028), and late disease stage (*P =* 0.035). However, these patients had no significant differences in developing SPMs, regarding sex (*P =* 0.170) and treatment (*P =* 0.111).

**Table 1:** Distribution of demographic and risk factors in the patient cohorts with index OPC and non-OPC

Variables	Total	OPC (*N =* 752)			Non-OPC (*N =* 777)		
	No.	SPM (N)	%	p*	SPM (N)	%	p*
Total patients	1529	42	5.6		82	10.6	
Age				0.004			0.024
≤ Median (57 years)	770	12	28.6		33	40.2	
> Median (57 years)	759	30	71.4		49	59.8	
Sex				0.546			0.170
Male	1186	35	83.3		62	75.6	
Female	343	7	16.7		20	24.4	
Tobacco smoking				0.010			0.037
Ever	1009	22	52.4		69	84.1	
Never	520	20	47.6		13	15.9	
Alcohol drinking				0.813			0.005
Ever	1099	30	71.4		69	84.1	
Never	430	12	28.6		13	15.9	
Ethnicity				0.598			0.028
Non-Hispanic White	1333	38	90.5		60	73.2	
Other	196	4	9.5		22	26.8	
Index cancer stage				0.240			0.035
I or II	386	5	11.9		26	31.7	
III or IV	1143	37	88.1		56	68.3	
Treatment^a^				0.511			0.111
S only	253	0	0.0		19	23.2	
S/C/X	335	4	9.5		30	36.6	
X/C	941	38	90.5		33	40.2	

*χ2 test for differences between patients with and without SPM

^a^S, surgery, C, chemotherapy, and X, radiation

### *FAS/FASL* polymorphisms and risk of SPM after index OPC and non-OPC

Table 2 shows both univariate and multivariable analyses on SPM risk associated with *FAS/FASL* polymorphisms after index OPC and non-OPC. For multivariable analysis, the estimates of associations were adjusted by age, gender, ethnicity, smoking and drinking status, index cancer stage, and treatment. The patients with OPC and *FASL*844 CT/TT genotypes were found to significantly increase the risk of SPM compared with those with the corresponding CC genotype (cHR, 2.5, 95 % CI, 1.1–5.8, *P =* 0.043 and aHR, 2.7, 95 % CI, 1.2–6.0, *P =* 0.032, respectively); however, such a significant association was not observed for other polymorphisms. In contrast, the non-OPC patients carrying *FAS*670 AG/GG and *FASL*844 CT/TT variant genotypes had approximately 2.5 times (cHR, 2.2, 95 % CI, 1.2–5.7, *P =* 0.048 and aHR, 2.4, 95 % CI, 1.1–5.1, *P =* 0.043, respectively) and 2.0 times (cHR, 1.8, 95 % CI, 1.1–3.2, *P =* 0.041 and aHR, 1.7, 95 % CI, 1.0–3.0, *P =* 0.049, respectively) increased risk of SPMs compared with those with the corresponding AA or CC genotypes, respectively.

**Table 2:** Genotype distribution of the *FAS* and *FASL* polymorphisms among patients with index OPC and non-OPC and their associations with risk of SPM

Variables	OPC (*N =* 42)		Non-OPC (*N =* 82)		cHRs^a^ (95 % CI), P		Adj. HRs (95 % CI) ^b^, P	
	SPM (N)	%	SPM (N)	%	OPC	Non-OPC	OPC	Non-OPC
*FAS 670 A > G*								
AA (ref.)	7	16.7	13	15.9	1.0	1.0	1.0	1.0
AG + GG	35	83.3	69	84.1	1.9 (0.8–4.9), 0.121	2.2 (1.2–5.7), 0.048	2.2 (0.9–5.3), 0.051	2.4 (1.1–5.1), 0.043
*FAS 1377 G > A*								
GG (ref.)	34	80.9	64	78.0	1.0	1.0	1.0	1.0
AG + AA	8	19.1	18	22.0	0.7 (0.3–2.1), 0.435	1.1 (0.4–1.6), 0.378	0.8 (0.4–1.9), 0.476	1.0 (0.5–1.8), 0.622
*FASL124 A > G*								
AA (ref.)	31	73.8	63	76.8	1.0	1.0	1.0	1.0
AG + GG	11	26.2	19	23.2	1.0 (0.4–2.7), 0.489	1.4 (0.7–2.8), 0.567	1.1 (0.5–2.4), 0.587	1.6 (0.9–3.0), 0.124
*FASL844 C > T*								
CC (ref.)	11	26.2	26	31.7	1.0	1.0	1.0	1.0
CT + TT	31	73.8	56	68.3	2.5 (1.1–5.8), 0.043	1.8 (1.1–3.2), 0.041	2.7 (1.2–6.0), 0.032	1.7 (1.0–3.0), 0.049

ref.: reference group

^a^Crude HRs

^b^HRs were adjusted for age, sex, ethnicity, smoking, alcohol, index cancer stage, and treatment

### Associations of the combined *FAS/FASL* variant genotypes with risk of SPMs after index OPC and non-OPC

As shown in Table 3, we performed the combined analysis of all 4 SNPs to focus on potentially modifying effect of the combined variant genotypes on risk of SPM since any of the 4 SNPs of *FAS* and *FASL* genes in the apoptotic pathway appeared to have a minor effect on risk of SPM. To perform the combined analysis of all 4 SNPs, we categorized all patients into 4 groups: (1) 0–1 variant genotype group; (2) 2 variant genotype group; (3) 3 variant genotype group; and (4) 4 variant genotype group based on the number of variant genotypes of the 4 polymorphisms. For the combined analysis, we found that the patients carrying 3 or 4 variant genotypes had an approximately 2-fold significantly increased risk of SPM compared with those with 0–1 variant genotypes only for non-OPC in univariate Cox analyses (cHR, 1.8 and 2.7, 95 % CI, 0.9–3.5 and 1.2–6.1, and *P =* 0.061 and 0.014, respectively) and multivariable Cox analyses (aHR, 1.8 and 2.2, 95 % CI, 1.0–3.9 and 1.2–6.5, and *P =* 0.051 and 0.021, respectively), while such significant associations were not found for OPC patients (Table 3).

**Table 3:** SPM risk associated with combined *FAS/FASL* variant genotypes after index OPC and non-OPC

No. variant genotypes	OPC	Non-OPC	cHRs^a^ (95 % CI), P		aHRs (95 % CI)^b^, P	
	SPM/SPM-free (42/710)	SPM/SPM-free (*n =* 82/695)	OPC	Non-OPC	OPC	Non-OPC
0-1 (ref.)	5/142	10/132	1.0	1.0	1.0	1.0
2	15/256	27/264	1.3 (0.4–3.5), 0.673	1.4 (0.6–2.7), 0.585	1.3 (0.5–3.7), 0.698	1.5 (0.5–3.3), 0.612
3	16/220	31/229	1.6 (0.6–4.1), 0.587	1.8 (0.9–3.5), 0.061	1.7 (0.5–3.8), 0.632	1.8 (1.0–3.9), 0.051
4	6/92	14/70	2.2 (0.9–6.8), 0.112	2.7 (1.2–6.1), 0.014	2.0 (0.9–6.6), 0.110	2.2 (1.2–6.5), 0.021

^a^Crude HRs

^b^HRs were adjusted for age, sex, ethnicity, smoking, alcohol, index cancer stage, and treatment

### Stratification analysis of the combined *FAS/FASL* genotypes with risk of SPM

In the stratified analysis by smoking and drinking status, to increase statistical power, we categorized the patients into 2 groups based on the number of combined variant genotypes of the 4 polymorphisms (1) low-risk group (carrying 0–2 variant genotypes) and (2) high-risk group (carrying 3–4 variant genotypes) shown in Table 4. As we expected, the OPC patients in high-risk group had significantly increased risk for SPMs compared with those in low-risk group only for never smokers (cHR; 18.9, 95 % CI; 1.3–330.2, and *P =* 0.032 and aHR; 20.0, 95 % CI; 1.2–327.0, and *P =* 0.028, respectively) but not for ever smokers (cHR; 1.4, 95 % CI; 0.5–3.3, and *P =* 0.722 and aHR; 1.4, 95 % CI; 0.6–3.4, and *P =* 0.787, respectively). No such significant associations were found for either ever drinkers or never drinkers among index OPC patients (Table 4). However, the non-OPC patients in high-risk group had an approximately 2-fold significantly increased risk of SPM compared with those in low-risk group in both ever smokers (cHR; 2.1, 95 % CI; 1.1–4.2, *P =* 0.013 and aHR; 2.3, 95 % CI; 1.2–4.4, and *P =* 0.011, respectively) and ever drinkers (cHR; 2.3, 95 % CI; 1.1–3.9, and *P =* 0.022 and aHR; 2.2, 95 % CI; 1.2–4.1, and *P =* 0.019, respectively), whereas such significant associations were not observed in either never smokers (cHR; 0.2, 95 % CI; 0.1–1.4, and *P =* 0.346 and aHR; 0.1, 95 % CI; 0.0–1.2, and *P =* 0.323, respectively) or never drinkers (cHR; 0.5, 95 % CI; 0.2–4.3, and *P =* 0.798 and aHR; 0.6, 95 % CI; 0.1–4.1, and *P =* 0.801, respectively).

**Table 4:** SPM risk associated with combined *FAS/FASL* variant genotypes after index OPC and non-OPC, stratified by smoking and alcohol

Variables	Low-risk group^a^		High-risk group^b^		cHRs^c^, (95 % CI), P	aHRs^d^ (95 % CI), P
	No.	%	No.	%	Low-risk group (ref.) *vs*. High-risk group	Low-risk group (ref.) *vs*. High-risk group
OPC	575	76.5	177	23.5		
Smoking						
Ever	325	56.5	104	58.8	1.4 (0.5–3.3), 0.722	1.4 (0.6–3.4), 0.787
Never	250	43.5	73	41.2	18.9 (1.3–330.2), 0.032	20.0 (1.2–327.0), 0.028
Alcohol						
Ever	421	73.2	128	72.3	1.3 (0.3–3.7), 0.823	1.4 (0.5–3.8), 0.822
Never	154	26.8	49	27.7	1.9 (0.3–17.2), 0.887	1.8 (0.2–16.1), 0.867
Non-OPC	614	79.0	163	21.0		
Smoking						
Ever	455	74.1	125	76.7	2.1 (1.1–4.2), 0.013	2.3 (1.2–4.4), 0.011
Never	159	25.9	38	23.3	0.2 (0.1–1.4), 0.346	0.1 (0.0–1.2), 0.323
Alcohol						
Ever	426	69.4	124	76.1	2.3 (1.1–3.9), 0.022	2.2 (1.2–4.1), 0.019
Never	188	30.6	39	23.9	0.5 (0.2–4.3), 0.798	0.6 (0.1–4.1), 0.801

^a^Low-risk group: 0–2 variant genotypes

^b^High-risk group: 3–4 variant genotypes

^c^Crude HRs

^d^HRs were adjusted for age, sex, ethnicity, smoking, alcohol, index cancer stage, and treatment

## Discussion

In the present study, we evaluated the associations between the functional *FAS/FASL* polymorphisms and the risk of SPMs among both index OPC *versus* index non-OPC patients. We found that these polymorphisms modified the risk of SPMs differently for index OPC from non-OPC patients. Furthermore, when we stratified the risk factors of smoking and drinking status for further analysis, the significant associations of *FAS/FASL* variant genotypes with the risk of SPM were observed within several subgroups. These results might suggest that genetic factors, within the context of previous or continued exposure to smoking and alcohol may affect the risk of SPM after index OPC and non-OPC patients. These results support growing evidence showing that apoptosis is associated with risk of SPMs; and tumor site factor may contribute to this association. Thus, apoptotic variants modify the risk of SPMs associated with the smoking and alcohol exposure in a tumor site-specific manner.

The roles of FAS and FASL in tumor formation have been well studied. The down-regulation of FAS may protect tumor cells from elimination by antitumor immune responses, whereas up-regulation of FASL may increase the ability of tumor cells to counterattack the immune system by inducing apoptosis of FAS-sensitive lymphocytes [30–33]. Therefore, alteration of the *FAS* and *FASL* expressions may decrease the apoptotic ability of cells, and many tumor cells may evade or suppress the immune system. Most of the studies suggested that decreased *FAS* gene expression and/or increased *FASL* gene expression is a common feature of malignant transformation and an early event associated with the development of human cancers [26, 34–42]. Survival analyses have shown that *FAS*1377 and *FAS*670 polymorphisms are associated with lower survival in early stage non-small cell lung cancer patients and increased risk of recurrence and death in epithelial ovarian cancer patients [38, 43]. These findings support the biological plausibility that the alteration of *FAS* or *FASL* genes, such as functional genetic polymorphisms, may affect the risk for tumor development, including SPM.

These four polymorphisms in gene promoters may affect function of *FAS* and *FASL* genes, and result in expression changes of these genes [26, 27, 42]. Change of *FAS* or *FASL* expression resulting from their promoter variants may help the transformed cells evade FAS-mediated cell death, subsequently affecting risk for SPMs. In the current study, we observed a significant association between *FAS*670 polymorphism with risk for developing SPMs among index non-OPC but not for OPC patients, while a significant association of *FASL*844 polymorphism with risk of SPMs among both index OPC and non-OPC patients. These findings might suggest that there were different etiologies between OPC and non-OPC patients underlying these observed associations. Although these polymorphisms are considered to be biologically functional, the findings on the association between risk of SPMs and these putatively functional polymorphisms were inconsistent. It might be likely that the study patients were stratified by the index tumor sites, which is a potential confounding factor when studying overall index SCCHN patients.

Previous studies suggested that the two *FAS* or *FASL* polymorphisms were not in a linkage disequilibrium and these polymorphisms had a joint effect on cancer risk [26], and the combined *FAS/FASL* genotypes were associated with a significantly increased risk of SCCHN. In the current study, such a significant association of the combined variant genotypes of these polymorphisms with the risk of SPM was found for non-OPC but not for OPC. The presence and absence of the joint effect among index OPC patients and index non-OPC might result from the different roles of *FAS/FASL* polymorphisms in etiology of SPM development through gene-gene or gene-environment (smoking, alcohol, HPV status, and other environmental risk factors) interactions in different site of SCCHN tissues.

Tobacco smoking, alcohol use, and HPV infection have been well-established as risk factors for SCCHN, particularly smoking/alcohol for non-OPC, and HPV for OPC. Several studies have reported such gene-environmental interactions on risk of SCCHN. A study on Taiwanese population reported that *FAS*1377 and *FAS*670 polymorphisms were strongly correlated with environmental factors like cigarette smoking, betel chewing, and alcohol drinking and had effects on increased risk of oral cancers [44]. Another study on Asian Indian population also found *FAS*1377, *FAS*670, and *FASL*844 polymorphisms interacted with tobacco smoking to increase the risk of oral cancers [45–47]. The interaction effect between smoking and *FASL*844 polymorphism on the risk was also observed in other types of cancer, including lung, pancreatic, and esophageal cancers [34, 36, 48, 49]. Our current study when stratified by smoking and alcohol status showed a consistency with the previous studies. The patients with index non-OPC, who had the combined variant genotypes and who were ever smokers and ever drinkers, had a significantly higher risk for SPMs, while the patients with OPC, who had the combined variant genotypes and who were never smokers, had a significantly higher risk for SPMs, but no such a significant risk was found among ever smokers. This might be because that index non-OPC are caused by smoking and alcohol exposure, whereas most of index OPC patients occurs in never smokers and are caused by HPV.

This is the first study to evaluate the associations between *FAS/FASL* polymorphisms and the risk for SPM among index OPC *versus* non-OPC patients. Although previous studies have found significant associations between the polymorphisms and SPM among the SCCHN patients, they did not consider index tumor site as an important confounding factor for such association due to different risk factors for OPC from non-OPC patients. Since OPC has distinguished epidemiological and clinical features along with prognosis that are different from non-OPC, it is important to investigate the associations stratified by the tumor sites. Therefore, this current study might provide novel information with clinician for secondary cancer prevention and improved prognosis of patients with primary SCCHN.

The results from such a stratified analysis by index tumor site in the present study is essential to improving long-term patient outcomes and accurate understanding the unique risk factors and carcinogenesis of SCCHN at particular subsites. In addition, the high frequency of SPM occurs in approximately 15 % of SCCHN patients. In patients with SCCHN, index cancer site, HPV status, and smoking use greatly affect the risk and distribution of SPM. Although the incidence of SCCHN in the U.S. has been in decline over past two decades and the diagnostic and therapeutic approaches for the patients have been improved, the poor prognosis for OPC and non-OPC patients has moderately and not significantly improved, respectively. Therefore, *FAS* and *FASLG* polymorphisms may serve as a marker for genetic susceptibility to SPMs after index HPV-associated OPC and non-HPV-associated non-OPC, and for identifying high-risk subgroups in each cohort who might benefit from management of alternative treatment and predictable patient outcome. Moreover, identifying markers of risk for SPM among cancer survivors is currently limited to rather simplistic clinical posttreatment screenings.

There are several limitations in the current study. Firstly, a selection bias likely exists for study patients due to the hospital-based nature of this study. Secondly, as the rate of SPM is low in the index patient populations, the numbers of SPM event are limited the statistical power in this study. After series of stratifications, the numbers in some of the stratum or subgroups are relatively small, thus our results may come by chance. Thirdly, the late stage patients may have limited time for follow-up, since those patients might not have enough time to develop SPMs. Thus, the limited follow-up time may bias our estimates of the associations. Furthermore, screening bias in detecting SPM’s is not fully ruled out, since tobacco-associated SPMs are usually easier to be detected than non-tobacco-associated SPM. And finally, due to the retrospective nature of the original study design, we did not have enough information on HPV infection and the continued smoking behavior after index patient diagnosis, and these potential confounders could also bias the observed associations.

## Conclusion

*FAS/FASL* variants may individually or jointly modify the risk of SPM in OPC and non-OPC differently, particularly in some subgroups. Further studies with larger sample size are warranted to validate our findings; and these large molecular epidemiological association studies also should be considered to incorporate HPV information and smoking behavior after index cancer diagnosis or treatment into the study design.

## Abbreviations

SCCHN: squamous cell carcinoma of the head and neck
SPM: second primary malignancies
HR: hazard ratio
95 % CI: 95 % confidence interval
HPV: human papillomavirus
OPC: oropharyngeal cancer
non-OPC: nonoropharyngeal cancer
